# Resolutions of Respect—Dr. W. H. Dwinelle

**Published:** 1896-06

**Authors:** 


### RESOLUTIONS OF RESPECT—DR. W. H. DWINELLE.

Whereas, in the death of Dr. W. H. Dwinelle the American Academy of Dental Science loses an esteemed Honorary Fellow, whose long and active life has been of unusual benefit to our profession; therefore be it

*Resolved*, That the American Academy of Dental Science hereby records its sense of sorrow in the death of Dr. W. H. Dwinelle, being a loss to our society as well as to our whole profession.

That to his family we extend our sincere and heartfelt sympathy.

That upon the records of our society the above be recorded in affectionate memory, and that a copy of this be sent to his family.

(Signed) T. G. W. Werner,
Eugene H. Smith,
Charles H. Taft,
*Committee.*

March 31, 1896.

